# Expressive Faces Confuse Identity

**DOI:** 10.1177/2041669517731115

**Published:** 2017-09-19

**Authors:** Annabelle S. Redfern, Christopher P. Benton

**Affiliations:** School of Experimental Psychology, 1980 University of Bristol, UK

**Keywords:** visual perception, face perception, facial identity, facial expressions

## Abstract

We used highly variable, so-called ‘ambient’ images to test whether expressions affect the identity recognition of real-world facial images. Using movie segments of two actors unknown to our participants, we created image pairs – each image within a pair being captured from the same film segment. This ensured that, within pairs, variables such as lighting were constant whilst expressiveness differed. We created two packs of cards, one containing neutral face images, the other, their expressive counterparts. Participants sorted the card packs into piles, one for each perceived identity. As with previous studies, the perceived number of identities was higher than the veridical number of two. Interestingly, when looking within piles, we found a strong difference between the expressive and neutral sorting tasks. With expressive faces, identity piles were significantly more likely to contain cards of both identities. This finding demonstrates that, over and above other image variables, expressiveness variability can cause identity confusion; evidently, expression is not disregarded or factored out when we classify facial identity in real-world images. Our results provide clear support for a face processing architecture in which both invariant and changeable facial information may be drawn upon to drive our decisions of identity.

Every time we look at someone’s face, we see a unique instance of it. This is because changes occur from one moment to the next, such as the direction of gaze, head angle and expression. Over longer time periods, there is yet further variation in appearance, from changes in aspects such as hairstyle, adiposity and age. Our face recognition system is challenged by variability, particularly when faces are unfamiliar to us (Jenkins, White, Van Montfort, & Burton, 2011); after all, it makes intuitive sense that we may fail to recognise someone we hardly know if their facial appearance has changed, such as from weight loss or growing a beard. However, exposure to extensive facial variability has been shown to assist in learning new faces (Andrews, Jenkins, Cursiter, & Burton, 2015).

To explore how identity discrimination can be affected by variability, Jenkins et al. (2011) used a card-sort paradigm. In the first experiment of this study, participants were tasked with sorting a pack of 40 cards – each depicting the image of a face – into a pile for each identity. These face images were naturalistic ‘ambient’ images (Jenkins et al., 2011) taken from the environment and therefore inherently variable. Although the card pack comprised images of only two different unfamiliar individuals, most participants sorted it into many more identity piles. This contrasts sharply with the performance of participants familiar with the identities who showed near-perfect performance (Jenkins et al., 2011).

The findings of Jenkins et al. (2011) demonstrate how trivially easy it is for our face recognition system to overcome variability when faces are known to us, and how hard the task is when faces are unknown. Clearly, variability in unknown faces leads to a failure to tell when those faces should be grouped together. Interestingly, the other side of that coin – telling when faces should not be grouped together – seems unaffected by variability. When looking at the internal consistency of the sorted piles, Jenkins et al. found that most piles comprised images of only one actor. Despite the wide variability of the ambient images used in this study, there were very few misallocation errors.

In the present study, we consider the effect on recognition of manipulating just one source of variability in facial appearance: expressiveness. Expressions are universal (Ekman & Friesen, 1971) and ubiquitous and can cause someone’s appearance to alter extensively. When a face is familiar, however, this does not seem to be a particular challenge; after all, we recognise people we know with ease, irrespective of the expression on their face (Bruce & Young, 1986). This observation has added weight to the suggestion that identity and expressions are processed independently. However, this semblance of separability may instead be due to stored identity representations that are robust to ambiguity arising from expressions. Therefore, by investigating identity recognition with *un*familiar faces – for which we have no stored representation – we can address the question of whether expressions affect identity processing.

The literature related to this question is equivocal. Facial expressions have been found to help, to hinder, and to not affect unfamiliar face identity tasks. For example, there is evidence that expressions facilitate facial discrimination learning (Lorenzino & Caudek, 2015); conversely, expressions were found to reduce accuracy of unfamiliar face recognition (Bruce, 1982; Bruce, Valentine, & Baddeley, 1987) and slow it (Van Den Stock & de Gelder, 2014); and some studies have shown no effect of expressions on unfamiliar face recognition (e.g. Kaufmann & Schweinberger, 2004). Expressions have been shown to not only impede identity tasks (e.g. Fisher, Towler, & Eimer, 2016) but also facilitate them (e.g. Levy & Bentin, 2008); and there is evidence suggesting an asymmetric relationship between the two, with identity affecting expressions but not affected by them (e.g. Schweinberger & Soukup, 1998).

This heterogeneity of findings is paralleled by the differing stances of face processing theories with regard to the relationship between expression and identity processing. Dual-route theories propose early bifurcation of the visual routes that process changeable facial aspects (which includes expressions) and more invariant aspects – so those related to stable characteristics such as identity (Bruce & Young, 1986; Haxby, Hoffman, & Gobbini, 2002, 2000). They do not readily permit interactions between these functions because a strict interpretation is that they advocate independence of the mechanisms involved.

A different theoretical position has emerged from studies using principal component analysis to explore the relationship between identity and expression processing. This approach was used to investigate the statistical properties of faces, in order to determine which principal components code identity and which code expressions; it found that some principal components code both (Calder, Burton, Miller, Young, & Akamatsu, 2001). Developing this further, Calder (2011) proposed a single representational system for face processing in which some dimensions code expressions, some code facial identity and others code both. Later bifurcation of the processing routes for changeable and invariant face aspects occurs after this coding stage.

Calder (2011) suggested that this framework is located in the inferior occipital gyrus and fusiform gyrus, observing that most neuroimaging investigations of both expression and identity perception (e.g. Ganel, Valyear, Goshen-Gottstein, & Goodale, 2005) show fusiform gyrus sensitivity to both facial properties. It is therefore analogous to the identity processing route of Haxby et al.’s (2002, 2000) model. Haxby’s model identifies the inferior occipital gyrus and posterior superior temporal sulcus as the route for processing the changeable aspects of faces. This, Calder suggested, is a dynamic route involved in multisensory integration, hence its activation by expressive faces.

Calder (2011) argued that his framework not only permits separability but also predicts interaction between identity and expressions. A consequence of such a framework is the integration of changeable aspects with the more structural, invariant aspects associated with identity – thus, expressions are a part of our mental representation of facial identity. Burton, Jenkins and Schweinberger (2011) also propose that idiosyncratic within-person variability is integral to the representation of facial identity. These authors argue that the incorporation of changeable aspects with the identity representation is essential for enabling our recognition system to overcome the challenge of within-person variability.

In this study, we explore the effect of facial expressiveness variability on the recognition of unfamiliar faces. We use ambient images to ask whether the variability of facial expressiveness affects identity discrimination. The two experiments that follow show that expressiveness leads to more misallocation errors: The piles into which identities were sorted were more likely to contain a mix of two actors when the images were expressive, than when they were not. We clearly demonstrate facial expression information being factored into decisions of facial identity.

## General Method

### Developing the stimuli databases

We developed a database of 546 ambient face images (Redfern & Benton, 2017) from which we selected the stimuli for this experiment. These ambient images are of the faces of two actors, Luigi Lo Cascio and Fabrizio Gifuni. Both actors have extensive filmographies and TV credits but are little known in the United Kingdom, and therefore ‘unfamiliar.’ The database images incorporate extensive variability, from different sources: external variability (such as lighting), variability from image capture (such as camera resolution) and variation specific to the actor (such as facial hair). They were taken from YouTube and from DVDs of 13 movies (made between 2002 and 2014). In a similar manner to Jenkins et al. (2011), images exceeded 150 pixels in height and showed faces free of occlusion. We cropped images to the portrait dimension 4:5 and sized them to 320 × 400 pixels. All faces were either frontal or partial view.

**Figure 1.**
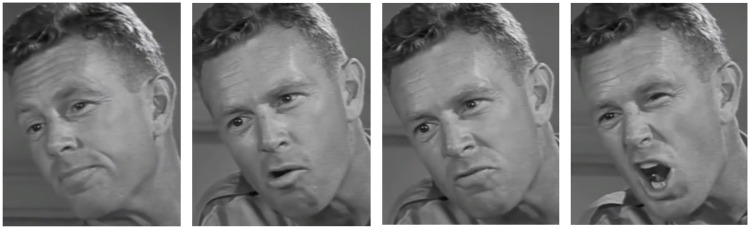
Example of a typical Image Group of images of actor Sterling Hayden, captured from the public domain movie ‘Suddenly’ (Bassler & Allen, 1954). Expressiveness ratings of the images are (from left): 35.0%, 52.5%, 68.5%, and 89.4%. These images provide an example of a typical Image Group and were not used in this study.

Images were gathered in ‘Image Groups’ – sets of two to nine face screenshots, all taken from the same scene and camera position. Copyright restrictions prevent us showing an Image Group; therefore, we have constructed one in Figure 1 for illustrative purposes. As is evident from this example, faces in the same Image Groups were similar (such as in image resolution, lighting direction, etc.) but differed in facial expressiveness.

We obtained expressiveness scores for each image, as follows: The 546 database images were printed in greyscale and laminated, to resemble small photographs. Participants (*n* = 40) who were unfamiliar with the actors, rating each image with a score ranging from 1 (‘neutral’) to 5 (‘very expressive’). From the totals of these scores, we calculated a mean for each image, which we rescaled to a percentage. Participants used their own judgement concerning the definition of neutrality and expressiveness; therefore, these terms relate to a layperson’s terminology. Our sample size of participants was speculative but similar to those of other facial images rating studies (e.g. 48 in Ebner, 2008; 24 in Palermo & Coltheart, 2004).

### Stimuli selection

**Figure 2.**
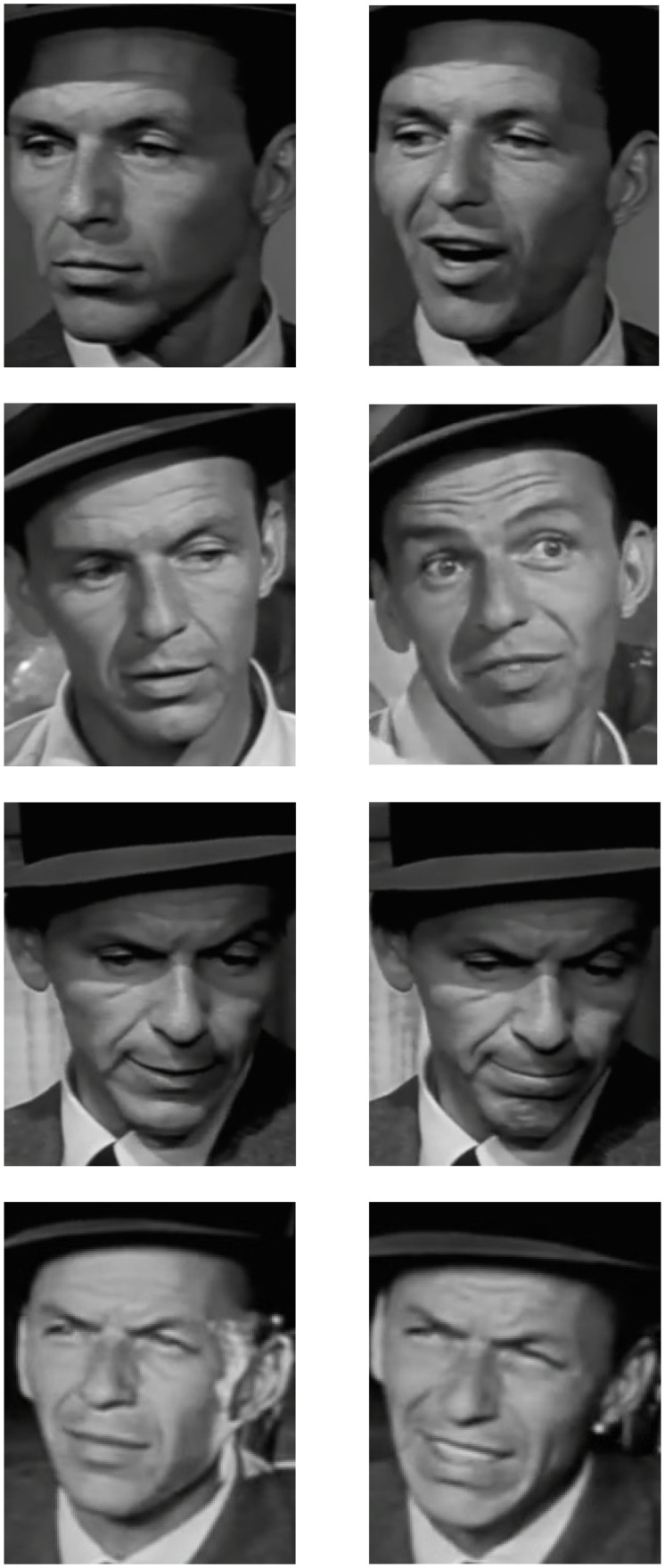
Example illustration of ‘neutral’ (left column) images and their ‘expressive’ counterparts (right column), all of the actor Frank Sinatra, taken from the motion picture ‘Suddenly’ (Bassler & Allen, 1954). Image pairs are each from a different Image Group. Expressiveness ratings are (from top, left to right): 11.3%, 56.9%, 26.3%, 58.8%, 37.5%, 52.5%, 38.1% and 60.0%. These images were not used in this study and are included for illustrative purposes.

We selected 40 pairs of images from the database, 20 of each actor. Each of these pairs was from a different Image Group, with no more than one pair selected from an Image Group. Importantly, each pair comprised one image that was low in expressiveness (i.e. <50% expressiveness score) and one that was high (i.e. >50%). These low and high expressive images formed the neutral and expressive card packs. Figure 2 illustrates this with example images, showing that for each card in the neutral pack, there was an expressive counterpart in the expressive pack. As these examples show, both the neutral and expressive sets of images contain substantial variability in, for example, variables such as lighting direction, camera, pixilation, age of actor, adiposity, skin texture, facial hair and so on. These variations are constant within pairs. The *only* difference between the conditions is therefore the variability of expressiveness that is greater in the expressive condition images than in the neutral images.

For the expressive card pack, the images we selected included a wide range of expressions and gesticulations. We took care to ensure a reasonable balance between negative and positive affect expressions (16:24 images), while not compromising the need for matching high and low expression images as closely as possible.

For our second experiment (see below), we created an additional ‘novel’ neutral pack for which we selected 40 images (20 of each actor) low in expressiveness. These were taken from Image Groups other than those used as the source of stimuli for the neutral and expressive packs, so as to ensure that they did not closely resemble those images. Table 1 shows the descriptive statistics of the selected stimuli.

**Table 1.** Descriptive Statistics of Stimuli Used in Experiments 1 and 2.

	Perceived expressiveness (%)			
Card pack type	Minimum	Maximum	Mean	±*SD*
Neutral^[1] [2]^	10.00	45.63	24.08	9.76
Expressive^[1] [2]^	53.75	100.00	80.27	12.02
Novel neutral^[2]^	6.25	27.50	18.00	5.16

*Note.* Numbers in square brackets denote the experiments that used these stimuli sets.

### Data analysis

We considered analysing our data using the technique introduced by Balas and Pearson (2017) in their card-sort paradigm. This approach quantifies errors within a signal detection framework. It does this by considering every possible pair of same identity pictures as signal and every pair of different identity pictures as noise. Any signal pairing within a sorted pile is a ‘hit,’ whilst every noise pairing is a ‘false alarm.’ Given that the total possible of each pairing is known, the proportions of hits and false alarms can readily be calculated, which means that *d*-prime and response criterion values can be derived from the card-sorting outcomes.

The method presents an elegant way of conceptualising and analysing results from card-sort tasks. We did not apply it to our data because our participants produced a substantial number of single-card piles, and we were unsure how to proceed (in Experiment 1, there were 217 such piles and 140 in Experiment 2). One of our reviewers noted that the presence of singletons should not preclude the use of Balas and Pearson’s technique, with singletons contributing to the same person in different group errors but not to different person in same group errors; we would welcome peer-reviewed confirmation of this point.

We did consider whether single-card piles were caused by a small number of problem images. If this had been the case, then these could have been simply taken out of the reckoning. Examination of the single-card piles shows that they were not attributable to a small number of images; instead, these piles comprised many of the images. For example, 28 of the 40 images in the Expressive pack were placed into single-card piles in Experiment 1.

Data were analysed by the number of identity card piles (veridical number being two) and by the internal accuracy of identity piles. We used these metrics to enable ease of comparison with other similar studies (Andrews et al., 2015; Jenkins et al., 2011; Neil, Cappagli, Karaminis, Jenkins, & Pellicano, 2016). We calculated inaccuracies as per Andrews et al. (2015): When piles contain images of both actors, cards depicting the least represented actor are recorded as misallocations. Thus, a card pile containing seven cards of Actor A and two of Actor B would score 2 misallocations, and a card pile containing six cards with three of each actor would score 3 misallocations.

Resultant data for both experiments had non-normal distributions that could not be normalised by transformation because of ceiling performance scores. Therefore, data were analysed by means of non-parametric permutation testing using code written in Matlab. Analyses were between-subjects and 100,000 permutations were conducted for each comparison. Because effect size cannot be meaningfully determined in a manner concomitant with our permutation tests, we report Cohen’s *d*; we also report *t*-tests to demonstrate that the results from non-parametric and parametric tests are similar. Error bars shown on all graphs indicate bootstrap-derived 95% confidence limits calculated using the percentile method (Efron & Tibshirani, 1994), in which within-person data were maintained so that we were not conflating within and between subjects variability.

## Experiment 1

Participants were tasked with sorting the neutral and expressive packs, one after the other, with order counterbalanced across participants. The task was to sort each pack into piles, with a pile for each perceived identity. Our primary interest was a between-participant comparison of the first card-sorting task, as this is the obvious comparison to test for an effect of expression on identity categorisation of unfamiliar faces. We included the second card sorts because we were also interested in the possibility of enhanced face learning from exposure to extensive within-person variability. This concept was demonstrated by Murphy, Ipser, Gaigg, and Cook (2015). Although this study did not specifically explore variability of expressiveness – therefore we cannot draw a direct comparison with the current study – it compared face learning in conditions of higher versus lower variability. Murphy et al. report superior face learning in their high variability condition; new identities were learned from a greater number of exemplars than in the low variability condition, while viewing time was equated. We are also interested in the related idea of ‘stability from variation’ (Bruce, 1994), where greater variability in face images of an individual can help define the boundary of possible images of that individual.

We reasoned that incorporating a second card sort into our experiment would enable us to test this. If variation confers a learning advantage, then participants should show particular improvement in the neutral-following-expressive condition, compared to the expressive-following-neutral condition. This is because, whilst the expressive pack encompasses the range of expression variability in the neutral pack, the reverse is not true. We did not plan comparisons between neutral first and neutral second nor between expressive first and expressive second, since we would be unable to assume whether any face learning was due to the neutrality or expressiveness of the faces, or the effect of practising the task.

### Method

#### Participants

We tested 85 naïve participants with a mean age of 20 years (range 18–35 years, 18 male). All were undergraduates who received course credit for their time. None were familiar with the actors whose images we used as stimuli, which was confirmed during debrief. Prior to this study, approval was obtained from the University’s Research Ethics Committee.

#### Stimuli

Stimuli comprised two packs of face image cards: a neutral pack and an expressive pack. Each pack contained 40 face images, 20 of each actor.

#### Procedure

The experiment was conducted in a quiet well-lit room. Participants were handed either the shuffled neutral or expressive card pack (the order of which was counterbalanced) and told that the pack contained the facial images of an unspecified number of males. Their task was to sort the pack into piles of cards, one pile for each person so that images of the same person were grouped together. Participants were given unlimited time to complete the task, as per the procedure of Jenkins et al. (2011). On completion, they were given a word search as a distractor task for 4 minutes or so, and then given the other card pack to similarly sort. Participant responses were recorded on scoresheets. Because each image had a unique image number, the scoresheets detailed which images were grouped together within the perceived identities.

### Results

**Figure 3.**
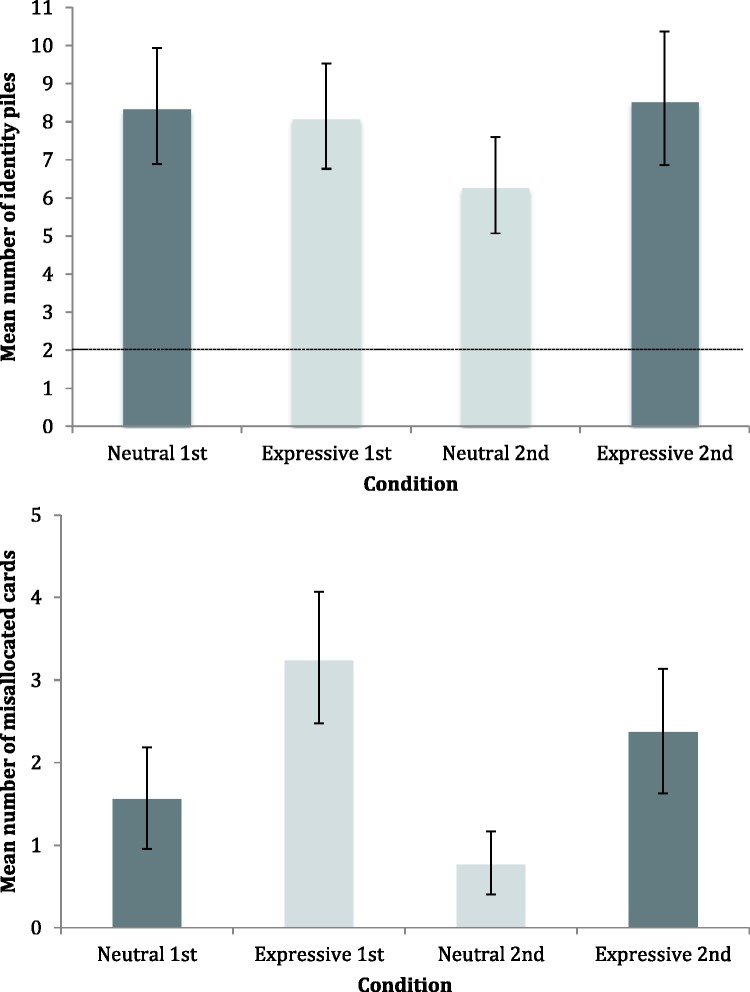
Results for Experiment 1. *Upper panel:* Mean number of identity piles per participant, by condition. Dotted line indicates veridical number of identity piles. *Lower panel:* Mean number of misallocated cards per participant, by condition. Shading indicates data from the two different participant groups, and error bars denote 95% confidence intervals.

Figure 3 (upper panel) shows the mean number of identity piles by condition. In all conditions, the packs were sorted into more perceived identities than the correct number of two. This difference is significant – the dotted line indicating veridical performance falls outside the 95% confidence limits for all four conditions (Cumming & Finch, 2005). As noted earlier, our primary interest was in the comparison of the neutral first and expressive first performance; however, as demonstrated by permutation test, *p* = .812 [*t*(83) = 0.24, *p* = .812, *d* = .05], there was no difference between these conditions.

For the second card sort, we measured superior performance in the neutral second condition, with permutation tests on the mean identity data showing this was significantly better than expressive second, *p* = .044 [*t*(83) = 2.01, *p* = .047, *d* = .44]. This indicates the possibility of learning; perhaps, sorting the expressive pack first enabled participants to better integrate the identities when they subsequently came to sort the neutral pack.

Figure 3 (lower panel) shows the mean number of misallocated cards by condition. We analysed these data to see if there was a difference in misallocations according to whether the images were neutral or expressive. Permutation tests revealed a difference between performance in the neutral first and expressive first card sorts, with participants making significantly more misallocation errors with the expressive images, *p* = .002 [*t*(83) = 3.24, *p* = .002, *d* = .70]. Indeed, in the expressive first card sort, 90.5% of the participants (38 of 42) made errors of this type, compared with 48.4% (21 of 43) in the neutral first card sort. This indicates that participants made comparatively more errors of mistaking one actor for the other when the faces were expressive – essentially, expressions lead to errors of telling which faces belong together.

Expressive pack misallocations were also evident in the second card sort. Permutation tests revealed a significant difference in performance between the neutral second and expressive second conditions, *p* < .001 [*t*(83) = 3.61, *p* < .001, *d* = .78], which shows that participants made more misallocation errors when they sorted expressive cards. Given that a similar difference exists between the first card sorts, this might simply be a repeat of that finding; it does not necessarily indicate learning from variability.

**Figure 4.**
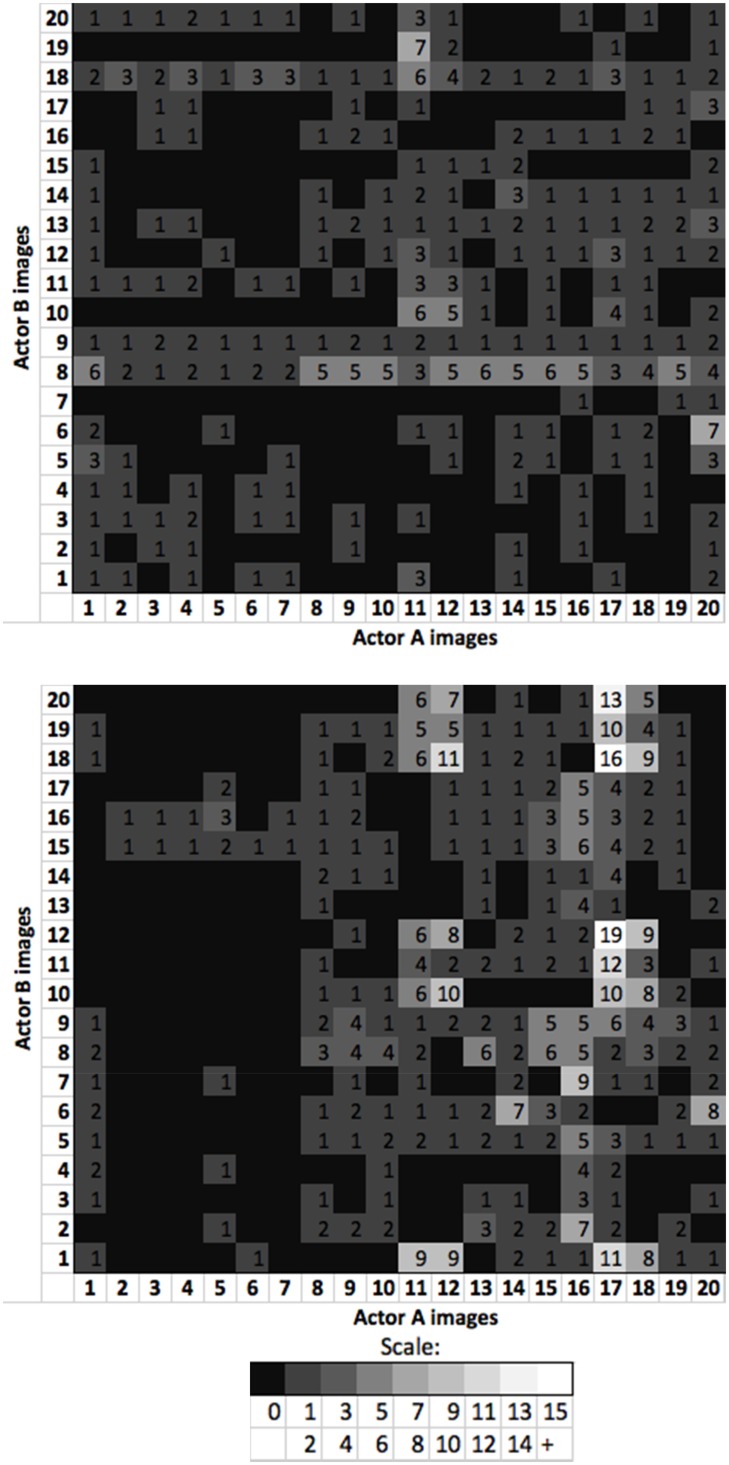
Experiment 1 misallocation matrices showing number of times each image of Actor A was misallocated with each image of Actor B. *Upper panel:* Matrix for neutral first condition. *Lower panel:* Matrix for expressive first condition.

We investigated the card misallocation data further to determine whether the misallocations were attributable to certain images or whether misallocations were widespread across the whole set of images in each pack. We constructed matrices to record the number of instances in our results, in which each image of Actor A was placed in an identity pile with an image of Actor B in the first card sorts. These are shown in Figure 4, in which the upper panel matrix shows the neutral first data and the lower panel matrix, the expressive first data.

The neutral first matrix (Figure 4, upper panel) shows that the misallocations are minimal, yet fairly widespread. Actor A Images 2 to 7 are rarely combined with Actor B images, which can be explained by the fact that these six images all feature a distinctive moustache that may have been a differentiating identity cue. Figure 4 (lower panel) shows that the expressive first condition misallocations were widespread across the image set and reveals that Actor A Image 17 is frequently misallocated with Actor B images.

In order to check that our misallocation results were not driven by the effects of Actor A Image 17, we removed it from our data and conducted our analysis again. The permutation test comparing expressive first and neutral first adjusted misallocations remained statistically significant, *p* = .017 [*t*(83) = 2.50, *p* = .014, *d* = .54]. Similarly, permutations to compare the expressive second and neutral second adjusted misallocations also remained significant, *p* = .006 [*t*(83) = 2.79, *p* = .007, *d* = .61].

The difference in number of identity piles for the second task supports the idea of stability from variation, since the superior performance is achieved by those who had initially sorted the expressive pack, which is the more variable of the two. However, we should note that drawing such a conclusion is problematic because we have the effect of two ‘training’ manipulations (expressive vs. neutral initial card sort), each measured on a different ‘test’ set (neutral vs. expressive second card sort). Therefore, we are unable to determine whether the difference in number of identity piles is a result of the difference in the first card sort, the difference in the second card sort, or an interaction between the two.

In the experiment that follows, we changed our design to enable us to make a direct assessment of any advantages conferred by stability from variation. As before, all participants performed an initial card sort with either the expressive or neutral pack. However, all performed the same second card sort, with a novel neutral pack. This design eliminates the problem noted earlier, which had resulted in participants being in different ‘training’ conditions but also different conditions at ‘test.’

## Experiment 2

Participants performed two card-sort tasks, the first with either the neutral or the expressive pack and the second with a pack of previously unseen ‘novel’ neutral images. As before, the neutral pack images encompass extensive variability (in factors such as orientation, lighting, camera, facial hair, etc.) but not in perceived expressiveness; and the expressive pack images encompass the same variability but with the *additional* variability in expressiveness.

### Method

#### Participants

Participants totalled 60 (16 male) of whom 59 were students and 1 was a University of Bristol staff member. Mean age was 21 years (range 18–33 years). Of the total, 16 participants received course credit, and the remainder were compensated £5 for their time. All were unfamiliar with the database actors, confirmed during debrief. Prior to this study, approval was obtained from the University’s Research Ethics Committee. This sample size gave us power of .84 (for a two-tailed test based on an alpha of .05 and the Experiment 1 effect size of *d* = .78).

#### Stimuli

Stimuli comprised the expressive and neutral card packs used in Experiment 1, and an additional novel neutral pack containing images from different Image Groups that those used to generate the neutral and expressive packs (Table 1).

#### Procedure

The experiment was conducted in a quiet, well-lit room. Participants were randomly assigned to either the neutral or expressive condition, and the procedure was as for Experiment 1, except that all participants sorted the novel neutral pack as their second task.

### Results

**Figure 5.**
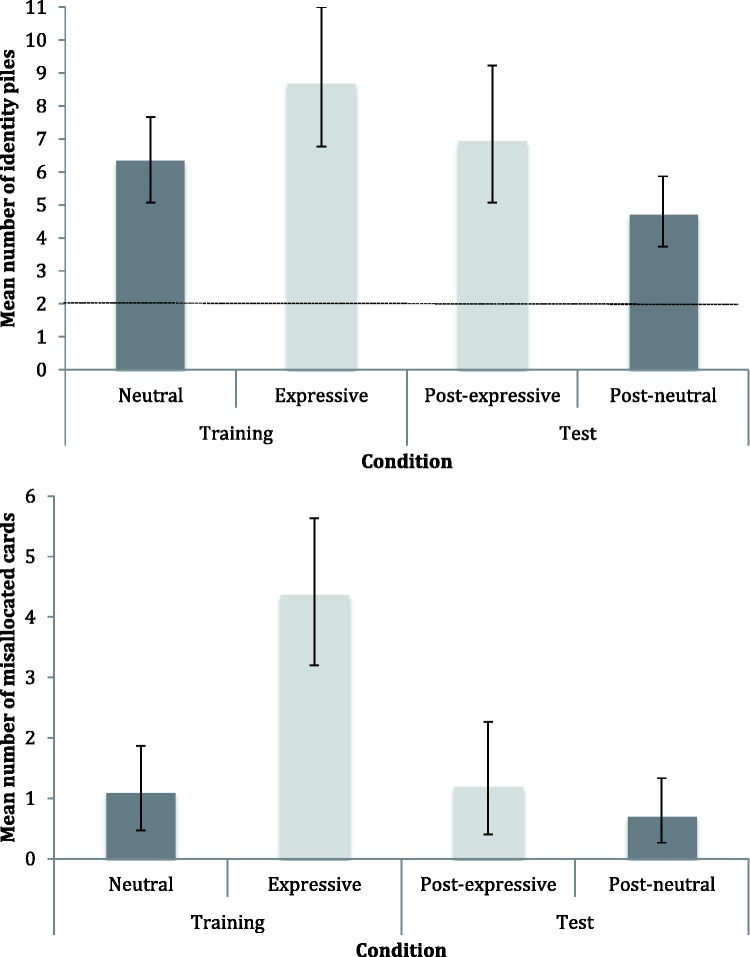
Results for Experiment 2. *Upper panel:* Mean number of identity piles per participant, by condition. Dotted line indicates veridical number of identity piles. *Lower panel:* Mean number of misallocated cards per participant, by condition. Shading indicates data from the two different participant groups, and error bars denote 95% confidence intervals.

Results are shown in Figure 5. As in Experiment 1, participants indicated significantly more perceived identities than the veridical number of two perceived identities than the veridical number of two (Figure 5, upper panel); (Cumming & Finch, 2005).

For the first card sort, the performance difference between conditions as measured by the identity number metric was not statistically significant, *p* = .068 [*t*(58) = 1.79, *p* = .079, *d* = 0.46]. However, performance between conditions as measured by the misallocation metric showed that there were significantly more misallocations with the expressive cards compared to neutral, *p* < .001 [*t*(58) = 4.47, *p* < .001, *d* = 1.16] (see Figure 5, lower panel).

In the second (‘test’) card sort with novel neutral images, permutation tests comparing the number of identities perceived in post-neutral and post-expressive conditions (Figure 4, upper panel) were not significant, *p* = .072 [*t*(58) = 1.83, *p* = .072, *d* = .47]. Permutation tests on the number of misallocations data indicated that test performance was not significantly different between conditions, *p* = .351 [*t*(58) = 0.89, *p* = .378, *d* = .23].

**Figure 6.**
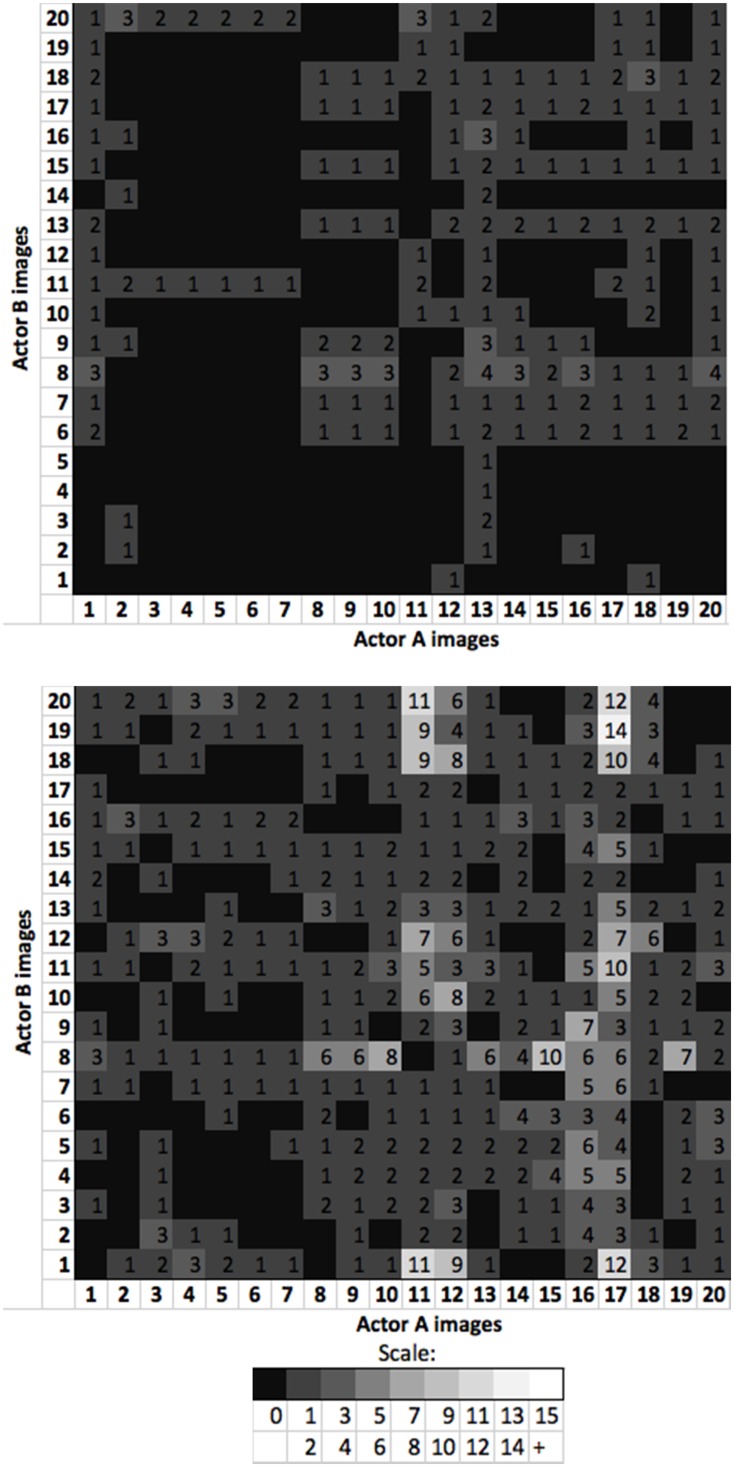
Experiment 2 misallocation matrices showing number of times each image of Actor A was misallocated with each image of Actor B. *Upper panel:* Matrix for neutral first condition. *Lower panel:* Matrix for expressive first condition.

As per Experiment 1, we created matrices to further explore the patterns of misallocated images between and across the image sets. We used the same approach as described earlier, and matrices for the first card sorts are shown in Figure 6.

The neutral first misallocations (Figure 6, upper panel) show that the neutral condition misallocations were widespread, albeit infrequent. Figure 6 (lower panel) shows that the expressive first misallocations were widespread across the images and, as for Experiment 1, Image 17 of Actor A was frequently combined within identity piles with Actor B images. To check that our results were not driven by the effects of this image, we removed the misallocation responses related to it and reanalysed our data. The permutation test comparing expressive first- and neutral first-adjusted misallocations remained statistically significant, *p* < .001 [*t*(58) = 3.75, *p* < .001, *d* = .97].

The result of the first (‘training’) card sort clearly shows significantly more errors of misallocation when faces were expressive compared to neutral. This repeats the findings of the initial card sort in Experiment 1 and strongly suggests that errors of when we mistake one person for another can be a consequence of increased expressiveness.

## General Discussion

We asked whether expressiveness would help or hinder identity classification of unfamiliar faces. To explore this question, we set participants the task of sorting cards – each depicting the face of an actor – into a pile for each identity. Both experiments show that participants were making two types of error: inferring more identities than there were; and mistaking two people as the same identity. We did not find a statistically significant difference between the numbers of perceived identities when the faces were expressive, as opposed to neutral. However, both experiments clearly demonstrate that more cards were misallocated when the task was performed with expressive faces than with neutral. Put another way, the errors of mistaking two people for the same person were present when the variable faces also varied in expressions and were negligible when the variable faces showed neutral expressions.

In the related literature, the error type of inferring too many identities has been termed failure to ‘tell faces together,’ and misallocations are termed failure to ‘tell faces apart’ (e.g. Andrews et al., 2015; Burton et al., 2011). In studies using the card-sort paradigm, the tendency has been for negligible errors of telling faces apart, compared to prevalent errors of telling faces together (e.g. Andrews et al., 2015; Jenkins et al., 2011). Indeed, a similar pattern has also been found in children. In a recent study that tested 4- to 12-year olds with a version of the card-sort paradigm that was modified for children, recognising an unfamiliar face despite appearance variability was found to be more challenging than discriminating between the two identities depicted on the cards (Laurence & Mondloch, 2016). What differentiates the findings of the current study is that we report substantial errors of ‘telling faces apart'.

There were a total of five neutral card sorts in our two experiments. All resulted in negligible card misallocations. The neutral face images in these packs varied substantially (in, e.g. lighting, orientation, camera, image quality, actor age, facial hair, hair style, adiposity, skin tone) but importantly, *not* in expressiveness. We can therefore conclude that variability in aspects, such as lighting and so on, of the images gives rise to only negligible misallocation errors.

In contrast, we found that misallocation errors were significantly greater in the three card sorts with expressive face images. The only difference between our neutral and expressive training packs is the expressiveness of the images – a deliberate feature of our experimental design, which ensures that each neutral pack image matches an expressive pack image in aspects other than expressiveness. Because of this, we can conclude that it is the variability in expressiveness that leads to misallocation errors.

However, a clear confound is whether the misallocation errors are attributable to an increase in the variability of expressiveness or the consequent increase in image variability. Clearly, the two aspects are inextricably linked in this study; an increase in the variability of expressiveness necessarily entails a corresponding increase in image variability (with all other image variables remaining constant across our matched image packs).

Turning to the related literature, several studies have compared face learning in conditions of high variability, with low variability; and they suggest that high variability helps, not hinders, face recognition. For example, a recent study by Ritchie and Burton (2017) concluded that exposure to high compared to low within-person variability led to superior learning of new identities. This study used as its low variability images, stills taken from interview videos of celebrities; its high variability images were sourced from Google Image searches.

The low variability images used by Ritchie and Burton (2017) do not include within-person variability in terms of lighting, hair style, age and adiposity – and are therefore considerably lower in variability than the neutral images in the current study. Indeed, the high variability images used by Ritchie and Burton (2017) more closely resemble the neutral images in the present study. The high and low variability distinction, therefore, differs between their study and ours. There are, therefore, clear stimulus differences between our study and those described earlier.

However, in another recent study, Murphy et al. (2015) used stimuli very similar to those in the experiments we report here. These authors found better recognition of novel test images of newly learned identities, from participants who had learned eight new faces in a high variability condition (from 96 ambient images of each face) compared to a low variability condition (from 6 repeated ambient images of each identity).

The findings of these studies – particularly of Murphy et al. (2015), given the similarity of their stimuli to ours – would suggest that higher image variability should *improve* performance. In the present study, we found that our higher variability, expressive images gave rise to more, not fewer, identity confusions than our lower variability, neutral images. So higher variability led to a *decrease* in performance. Therefore, we suggest that variability of expressions, and not image variability, is the likely cause of the identity confusions. Later in this discussion, we present a separate case for why expressions may be processed differently to other changeable aspects of faces.

Our finding of few misallocation errors in our neutral card sorts is mirrored in other studies that test adults with Caucasian face images using the same paradigm. For example, Jenkins et al. (2011) found that people allocated images to too many identity piles, but these identities were nonetheless internally consistent; and Andrews et al. (2015) demonstrated the same pattern in their results, as did Balas and Pearson (2017). Another study using this card-sort paradigm, but with typically developing and autistic children as participants, was conducted by Neil et al. (2016). Results of the adult sample, tested by Neil et al. (2016) for comparison purposes, follow the same pattern: significant errors with number of identities inferred but negligible misallocated cards.

Why then do these card-sort studies show levels of performance similar to our neutral card sorts? The card packs used by Jenkins et al. (2011), Andrews et al. (2015) and Neil et al. (2016) included facial expressions only incidentally, and the range of expressons was comparatively narrow. For example, the images used by Neil et al. (2016), illustrated in their paper, are low in expression variability (only neutral or smiling). And while those used by Jenkins et al. (2011), Andrews et al. (2015) and Balas and Pearson (2017) cannot be viewed because of copyright restrictions, an Internet search of the individuals they used – which is how they selected their images – reveals that these faces are also limited in variability of expression (again, only neutral or smiling). It seems likely, then, that the variability of the expressiveness of the ambient faces used in these studies was comparatively low. The pattern described earlier is consistent with our observation that facial image variations, from sources *other* than expressions, do not cause substantial misallocation errors.

This contrasts with our finding that variable expressiveness leads to identity misallocations, which is supported by neurological patients having been observed to make errors of mistaken identity when expressions change. For example, in a case study of face processing following amygdalotomy, the patient’s evident deficits in expression processing sometimes led her to interpret different expressions as different identities (Young, Hellawell, Van de Wal, & Johnson, 1996). As the amygdala is associated with emotion processing (Adolphs, Tranel, Damasio, & Damasio, 1994) and with supporting the recognition of different facial cues (Haxby et al., 2002, 2000), damage to this region is consistent with disruption to these processes, which is what we see in this patient.

Another neurological example is described by Etcoff (1984). She tested a group of 12 patients with right cerebral hemisphere damage and, in an initial identification task, 10 of these patients made errors in which they discriminated two facial identities as being three or four different identities when expression changed (Etcoff, 1984). Young et al. (1996) point out that both the patient they describe and those individuals described by Etcoff (1984) have difficulty in treating expressions and facial identity as independent sources of information.

The confusion between expressions and identity reported by Etcoff (1984) is starkly contrasted by Young et al.’s (1996) finding that the amydalotomy patient had unimpaired recognition performance when variable aspects of appearance *other* than expression were manipulated – variables such as lighting, orientation, facial hair and hairstyles. As noted earlier, the neutral card sort of our study and the card sorts of other studies (Andrews et al., 2015; Jenkins et al., 2011; Balas & Pearson, 2017; the adult sample in Neil et al., 2016) resulted in very few misallocation errors, despite the fact that all of these tasks incorporated substantial changes in non-expressive cues, such as lighting and viewpoint. These strands of evidence hint at a difference between the way our face processing system deals with variability from expressions and variability from other sources.

How might expression variability differ from variability from other sources? An important distinction is that expressions are specific to faces, whereas variations arising from sources, such as viewpoint or lighting, are common to all objects. This raises the possibility that this non-specific variability may be dealt with generic mechanisms that discount changes in viewing conditions to facilitate constancy (e.g. Gilchrist et al., 1999; Land & McCann, 1971; Marr & Nishihara, 1978; Sperandio & Chouinard, 2015; Tarr & Pinker, 1989). In contrast, expression variability is likely dealt with by a different, face-specific mechanism.

Furthermore, expressions are not only specific to faces but, to an extent, to *individual* faces (Burton, Kramer, Ritchie, & Jenkins, 2016). The underlying structure of an individual’s face will determine the particular characteristics of their expressions, so the ways in which facial expressions vary for any individual are therefore unique (Ganel & Goshen-Gottstein, 2004). To achieve expression-invariant face recognition would require learning the ways in which a particular face varies with expressions. Other sources of variability specific to faces include adiposity, ageing and facial hair; but as these aspects have a tendency to change incrementally over long time periods, a special mechanism to cope with their variability seems unlikely, beyond updating a stored representation in memory of how someone looks.

We have considered the view that our study may not generalise because it uses images of only two different people. Our investigation was, however, on within-person variability and not between-person variability. Therefore, an essential feature of our design was to use many images of few people, not few images of many people. The use of ambient images of a pair of individuals may give rise to effects that are particular to those faces, and not necessarily to others. Replication or quasi-replication is clearly warranted, and we would welcome investigations using this paradigm, ideally using ambient images of more individuals. Other studies using ambient images in card sorts mostly use faces of only two individuals (e.g. Andrews et al., 2015; Jenkins et al., 2011; Laurence, Zhou, & Mondloch, 2016; Neil et al., 2016), and the fact that these have all replicated the initial finding of Jenkins et al. (2011) – that image variability increases perceived number of identities – would suggest that this approach produces replicable outcomes. However, caution is warranted; we should look for both replication and convergence.

The expressiveness-dependent errors of mistaking two people for one in the present study clearly indicate that expression information is being used in identification judgements. We suggest that this is because the participants were misattributing expressiveness information as an identity signal. This result can be accommodated by Haxby et al.’s (2000) face perception model, which suggests that the fusiform face area may support expression perception because some expressions are idiosyncratic. Within this framework, facial expressions could be misinterpreted as identity cues when faces are unfamiliar, since the characteristics particular to identity and those particular to idiosyncratic expressions have yet to be learned. This explanation can also be applied to the face processing model proposed by Calder (2011), which suggests that the visual form of facial expressions and identity are coded in a single representational framework, before representations are processed further in other areas in the extended face processing network. Calder’s (2011) framework specifically predicts that expressions and identity will interact, and this fits with our finding that expressions interfere with identification when faces are unfamiliar.

The use of expression information in identification judgements is compatible with concepts of face representation proposing that expressions are an intrinsic part of facial identity. Therefore, learning a facial identity necessarily entails learning the range of ‘possible and permissible’ variability associated with that face (Bruce, 1994; Vernon, 1952). Burton et al. (2011) propose that idiosyncratic facial variability – including expressions – is essential for the development of stable identity representations. Burton et al. (2016) emphasise the importance of variability for learning *how* a face can vary and, therefore, the range of possible images of a particular individual.

What information we extract from faces and use to recognise them is probably the most important question in face processing research (Hole & Bourne, 2010). The answer has proved elusive, in part because the information we use and how we use it is modulated by our familiarity with the facial identity we are attempting to recognise. By this reasoning, expressions will help or hinder depending on familiarity. In the present study, we demonstrate that when faces are *un*familiar, variable expressions can *impede* recognition – which we suggest is because we have not yet learned the idiosyncratic variability associated with those faces. That typical expressions have been shown to *facilitate* recognition with *familiar* faces (e.g. Kaufmann & Schweinberger, 2004) demonstrates a different role for expressions, consistent with the idea that the idiosyncratic expressions can be used as an identification cue when faces are familiar (Ganel & Goshen-Gottstein, 2004).

In conclusion, both experiments reported here provide strong evidence that variability of expressions can lead to errors of mistaking two people as the same identity. To the best of our knowledge, the present study uniquely demonstrates this with neurologically normal adults. Our face images featured extensive within-person variability, yet it was expressiveness variability, over and above other variable aspects, that led to this type of error. This suggests that expressions may be processed in a fundamentally different way to other variable aspects of facial appearance. Finally our demonstration, that facial expressiveness leads to confusions of identity, is clearly supportive of theoretical approaches in which expression information can be readily and routinely incorporated into our processing of facial identity.
